# Asclepiasterol, a novel C21 steroidal glycoside derived from *Asclepias curassavica*, reverses tumor multidrug resistance by down-regulating P-glycoprotein expression

**DOI:** 10.18632/oncotarget.8965

**Published:** 2016-04-25

**Authors:** Wei-Qi Yuan, Rong-Rong Zhang, Jun Wang, Yan Ma, Wen-Xue Li, Ren-Wang Jiang, Shao-Hui Cai

**Affiliations:** ^1^ College of Pharmacy, Jinan University, Guangzhou, 510632, P. R. China; ^2^ Department of Toxicology, Guangzhou Center for Disease Control and Prevention, Guangzhou, 511430, P. R. China

**Keywords:** asclepiasterol, steroid, multidrug resistance (MDR), P-glycoprotein, phosphorylation of ERK1/2 (P-ERK)

## Abstract

Multidrug resistance (MDR) mediated by P-glycoprotein (P-gp) is a major cause of cancer therapy failure. In this study, we identified a novel C_21_ steroidal glycoside, asclepiasterol, capable of reversing P-gp-mediated MDR. Asclepiasterol (2.5 and 5.0μM) enhanced the cytotoxity of P-gp substrate anticancer drugs in MCF-7/ADR and HepG-2/ADM cells. MDR cells were more responsive to paclitaxel in the presence of asclepiasterol, and colony formation of MDR cells was only reduced upon treatment with a combination of asclepiasterol and doxorubicin. Consistent with these findings, asclepiasterol treatment increased the intracellular accumulation of doxorubicin and rhodamine 123 (Rh123) in MDR cells. Asclepiasterol decreased expression of P-gp protein without stimulating or suppressing *MDR1* mRNA levels. Asclepiasterol-mediated P-gp suppression caused inhibition of ERK1/2 phosphorylation in two MDR cell types, and EGF, an activator of the MAPK/ERK pathway, reversed the P-gp down-regulation, implicating the MAPK/ERK pathway in asclepiasterol-mediated P-gp down-regulation. These results suggest that asclepiasterol could be developed as a modulator for reversing P-gp-mediated MDR in P-gp-overexpressing cancer variants.

## INTRODUCTION

Successful cancer chemotherapy is limited by the development of resistant cancer cell variants. Some cancers develop resistance to individual cytotoxic drugs by altering the drug targets, while others can be resistant to many drugs with different chemical structures and unrelated mechanisms of action [1]. This phenomenon is called multidrug resistance (MDR), and it is the major obstacle of chemotherapy in cancer treatment. The underlying mechanisms responsible for MDR in cancer cells are complicated and still not completely understood. Many different mechanisms of MDR have been elucidated in recent years, including alterations in cycle checkpoints, failure of apoptotic mechanisms, repair of damaged cellular targets, and reduced drug accumulation [2].

Among these mechanisms, reduced drug accumulation in cancer cells caused by the over-expression of ATP-binding cassette (ABC) transporters has been studied in the most detail and appears to be a very common cause of MDR [1]. These transporters pump a wide range of structurally and functionally diverse amphipathic anticancer drugs out of tumor cells [3], resulting in failure of chemotherapy due to low intracellular drug concentrations. To date, 48 different ABC transporters have been identified in the human genome and divided into seven different classes (A-G) based on sequence similarity [4]. Three members of these transporters (B, C and G), such as P-glycoprotein (P-gp/ABCB1), breast cancer resistance protein (BCRP/ABCG2), and multidrug resistance-related protein1 (MDR1/ABCC1), confer drug resistance by pumping out a variety of anticancer agents from cells [5–7].

Research on ABC-transporter-mediated MDR has focused on P-gp [8]. P-gp is a classical ABC transporter that is overexpressed in many human solid and hematologic cancers [9], and is a confirmed marker of chemo-resistance and decreased survival in leukemia [10], lymphoma [9, 11], osteosarcoma [12], small-cell lung cancer [13] and breast cancer [14], Therefore the development of P-gp inhibitors is very important for chemotherapy. Many P-gp inhibitors such as valspoder (PSC-833), dofequidar fumarate (MS-209), tariquidar (XR9567) and thiosemicarbazone derivative (NSC73306) have been found to antagonize P-gp function both *in vitro* and *in vivo* [15–18]. However, phase III trials of these agents have been disappointing and none have achieved significant survival benefits [19, 20]. Thus, searching for new MDR modulators with higher efficacy and low toxicity is warranted.

Throughout history, humankind has used natural products from plants, animals, and microorganisms to treat diseases [21]. Even now, 80% of the world's population uses herbal medicines and increasing attention is being paid to natural products [22, 23]. Although most of the established resistance-modifying agents (RMA) are synthetic compounds and are toxic at the required dose, the search for P-gp inhibitors from natural products may be an alternative approach [24]. For example, natural products schisandrol A, tetramethylpyrazine, tetrandrine, and 23-hydroxybutulinic acid [25, 26] inhibit P-gp, indicating that searches for other natural products capable of modulating P-gp might be fruitful.

Steroids comprise a group of cyclic organic compounds characterized by a four-ring carbon structure. These compounds have been the focus of drug discovery not only because of their fascinating structures related to endogenous hormones, but also due to their diverse array of pharmacological activities [27]. Some steroids demonstrate intriguing anticancer properties [28]. For example, clinical trials of exemestane, a steroidal aromatase inhibitor, have shown advantages over non-steroidal aromatase inhibitors against breast cancer [29]. As another example, the growth and function of the prostate is dependent on androgens [30]. Potent and selective inhibition of CYP17A1 by abiraterone depletes residual non-gonadal androgens and is an effective treatment for castration-resistant prostate cancers [31]. Recently, many studies have pointed out that steroidal compounds can reverse MDR in cancer cells; however, the underlying mechanism of MDR-reversal by steroidal compounds remains unknown.

**Scheme 1:**
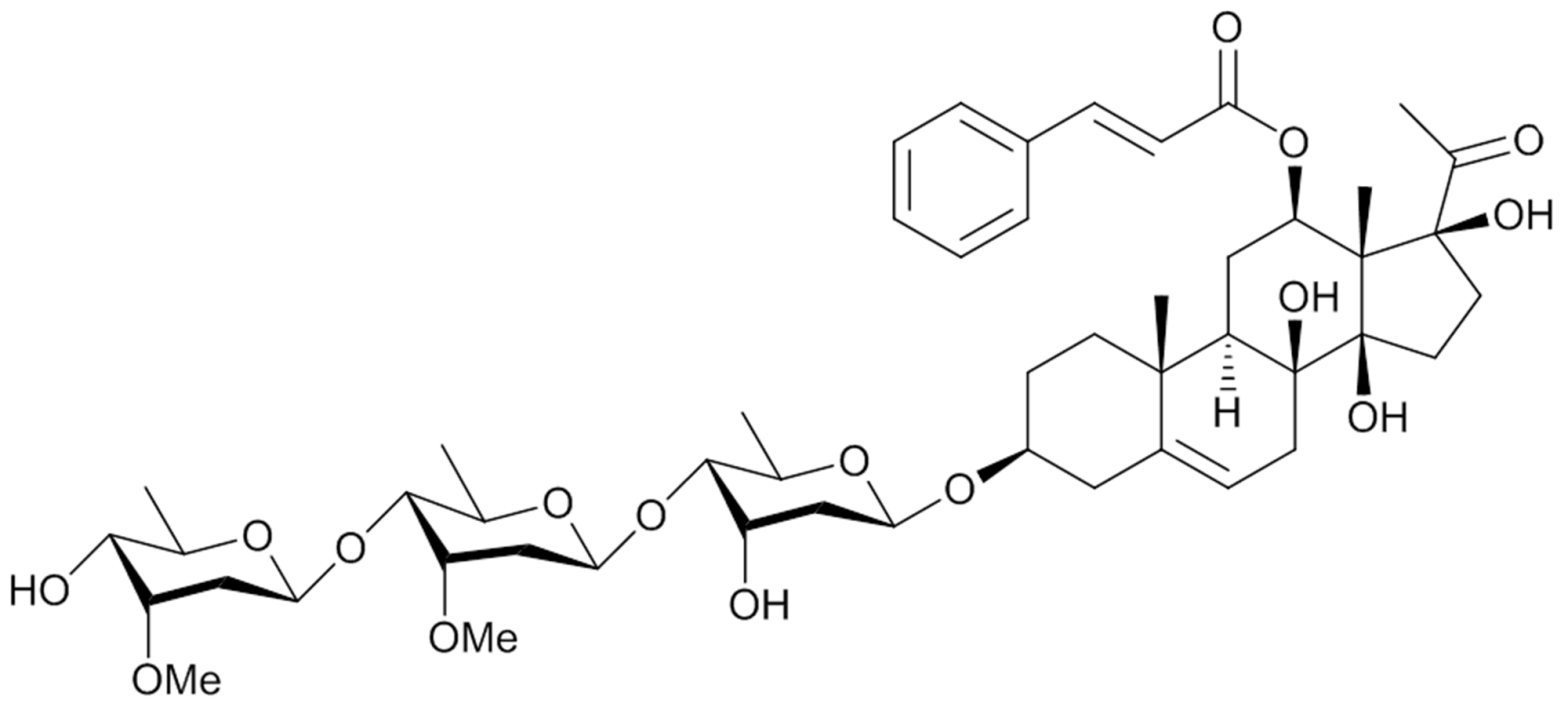
Chemical structure of asclepiasterol

In our previous study, we systematically examined the chemical constituents of *Asclepias curassavica*, a milkweed plant and a source of food for butterflies. We performed an extensive screen for P-gp-mediated MDR reversal and anticancer activities of these compounds [32]. We identified a novel steroidal compound asclepiasterol (Scheme 1), capable of reversing P-gp-mediated MDR. In the current study, we investigated the effects of asclepiasterol on P-gp-mediated MDR in P-gp-overexpressing cancer cells (MCF-7/ADR, HepG-2/ADM) and elucidated the underlying mechanisms. To our knowledge, asclepiasterol is the first C_21_ steroid glycoside found to decrease P-gp expression and reverse P-gp-mediated MDR.

## RESULTS

### Analysis of drug resistant profiles in MDR cells

**Figure 1:**
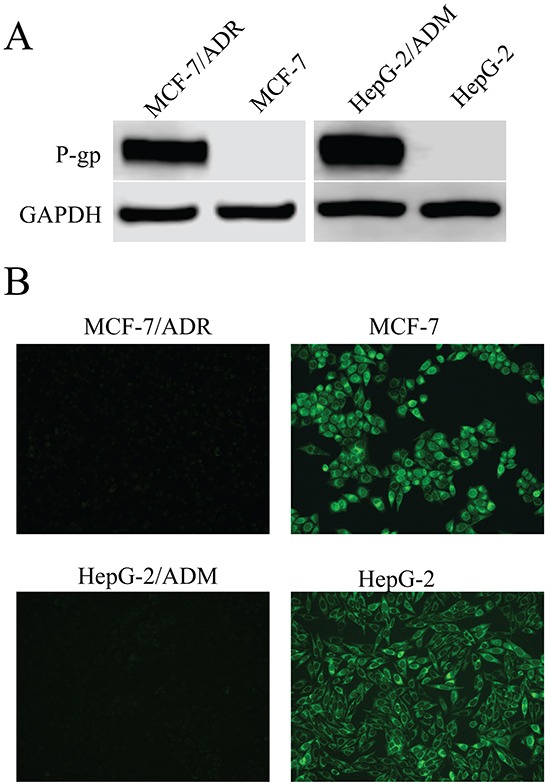
P-glycoprotein (P-gp) expression in MCF-7/ADR and HepG-2/ADM cells in comparison to corresponding parental cell lines, MCF-7 and HepG-2 **A.** Western blot analysis of proteins extracted from MDR cells and their parental cells with P-gp antibody. GAPDH was used as loading control. **B.** Fluorescence microscope detection of the accumulation of rhodamine123 (Rh123) in MDR cells and their parental cells. Images were acquired at 488nm extraction and 535nm emission wavelengths for Rh123.

We first examined the cytotoxic effects of several anticancer agents on MDR cells (MCF-7/ADR and HepG-2/ADM) and their parental cells. As shown in Table 1, the MDR cells showed significant resistance to these agents with 14.47, 17.62, 199.91, 442.42 multidrug resistance ratios (MRs) in MCF-7/ADR cells, and 108.94, 79.87, 116.74, 74.63 in HepG-2/ADM cells for doxorubin (Dox), epirubicin (EPI), daunorubicin (DNR) and paclitaxel, respectively. Since these anticancer agents are transported by P-gp [33], we measured the expression of P-gp in these cells and found that both MCF-7/ADR and HepG-2/ADM cells expressed higher levels of P-gp as compared with their parental cells (Figure 1A). Consistent with high P-gp expression, the intracellular accumulation of Rh123 (a specific fluorescent probe substrate for P-gp) in MDR cells was dramatically lower than in parental cells (Figure 1B, quantitative Rh123 accumulation results are shown in supporting information, Supplementary Figure S2). These results suggested that P-gp was overexpressed in MCF-7/ADR and HepG-2/ADM cells, which was consistent with our previous study [34]. Thus these two MDR cells were suitable target cells for studying the P-gp-mediated MDR reversal capability of asclepiasterol.

**Table 1:** Effect of asclepiasterol on reversing ABCB-1/P-gp mediated drug resistance

Compounds		IC_50_±SD^a^ (μM) (fold-reversal)^b^			
		MCF-7	MCF-7/ADR	HepG-2	HepG-2/ADM
Doxorubicin		0.995±0.012 (1.00)	14.401±2.811 (1.00)	0.430±0.019 (1.00)	46.856±8.244 (1.00)
+	2.5μM asclepiasterol	1.012±0.011 (1.02)	1.240±0.008 (11.61)**	0.429±0.020 (1.00)	4.584±0.027 (10.22)**
+	5.0μM asclepiasterol	1.022±0.012 (1.03)	0.411±0.010 (35.06)***	0.404±0.021 (0.94)	0.751±0.019 (62.41)***
+	10.0μM VRP	0.709±0.013 (0.71)	0.415±0.007 (34.71)***	0.137±0.189 (0.32)	1.219±0.024 (38.44)**
Epirubicin		4.282±1.202 (1.00)	75.466±9.523 (1.00)	3.556±0.852 (1.00)	283.973±12.254 (1.00)
+	2.5μM asclepiasterol	3.578±0.984 (0.84)	10.988±1.254 (6.87)**	3.088±1.258 (0.87)	21.268±4.579 (13.35)**
+	5.0μM asclepiasterol	3.540±0.842 (0.83)	4.528±1.362 (16.67)***	2.649±0.522 (0.75)	8.483±2.478 (33.48)***
+	10.0μM VRP	3.780±0.942 (0.88)	15.003±3.547 (5.03)*	1.210±0.014 (0.34)	38.234±9.547 (7.43)*
Daunorubicin		0.203±0.012 (1.00)	40.522±5.214 (1.00)	0.185±0.001 (1.00)	21.586±5.247 (1.00)
+	2.5μM asclepiasterol	0.260±0.022 (1.28)	1.051±0.987 (38.55)**	0.151±0.014 (0.82)	1.503±0.125 (14.36)**
+	5.0μM asclepiasterol	0.215±0.012 (1.06)	0.242±0.012 (167.31)***	0.192±0.051 (1.04)	0.889±0.128 (24.29)***
+	10.0μM VRP	0.234±0.531 (1.16)	0.694±0.052 (3.42)**	0.145±0.045 (0.78)	0.381±0.095 (56.69)**
Paclitaxel		0.239±0.091 (1.00)	105.738±12.345 (1.00)	0.101±0.012 (1.00)	7.531±1.242 (1.00)
+	2.5μM asclepiasterol	0.318±0.114 (1.33)	5.082±1.475 (20.81)**	0.105±0.009 (1.04)	0.117±0.009 (64.42)**
+	5.0μM asclepiasterol	0.262±0.051 (1.10)	0.370±0.095 (285.70)***	0.102±0.022 (1.01)	0.089±0.009 (85.09)***
+	10.0μM VRP	0.253±0.042 (1.06)	8.969±2.547 (11.79)**	0.099±0.003 (0.98)	0.096±0.003 (78.12)***
Cisplatin		39.881±2.515 (1.00)	37.685±0.395 (1.00)	40.019±1.267 (1.00)	30.937±1.439 (0.77)
+	2.5μM asclepiasterol	38.687±0.650 (0.97)	40.039±2.239 (0.94)	43.053±1.413 (1.07)	30.331±0.637 (0.76)
+	5.0μM asclepiasterol	38.792±1.080 (0.97)	38.726±0.706 (0.97)	42.477±3.498 (1.06)	31.213±1.573 (0.78)
+	10.0μM VRP	39.127±1.356 (0.98)	39.755±1.357 (0.95)	41.087±1.752 (1.02)	32.083±1.679 (0.80)

a Data in the table are shown as the means ± SD (n=6) of at least three independent experiments.

b The fold-reversals are calculated as RR value indicating the fold MDR reversal of inhibitors.

* *P* < 0.05

** *P* < 0.01

*** *P* < 0.001 for the IC_50_ versus that in the absence of inhibitors.

### Asclepiasterol is not cytotoxic to MDR tumor and non-tumor cells

**Figure 2:**
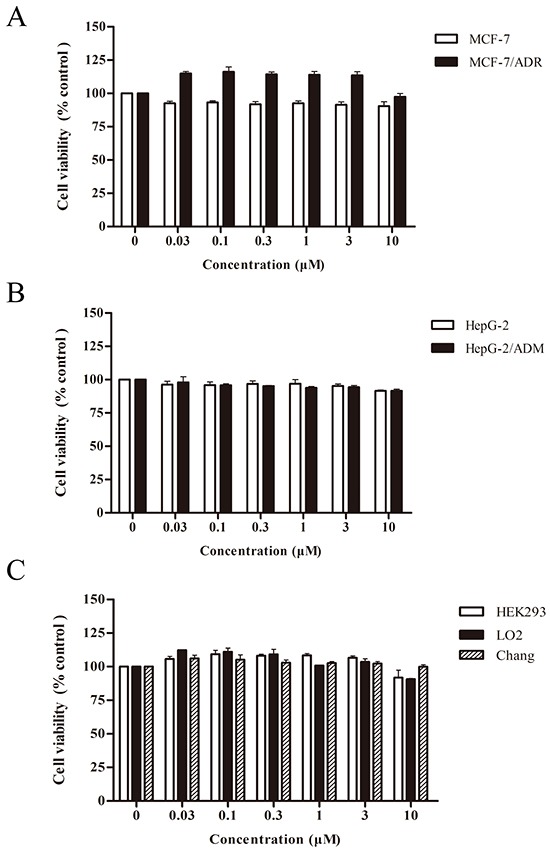
Cytotoxic effects of asclepiasterol The MTT cytotoxicity assay was assessed in pairs of multidrug resistant cell lines and their corresponding parental cell lines, as well as in non-tumor cell lines. Cells were treated with or without increasing concentrations of asclepiasterol for 48h and cell viability was determined. **A.** MCF-7 and MCF-7/ADR cells. **B.** HepG-2 and HepG-2/ADM cells. **C.** HEK293, LO_2_ and Chang cells. Inhibition of asclepiasterol on cell proliferation was calculated based on the absorbance ration between treatment and control. Values represent mean ± SD (n=6) of three independent experiments.

We next tested the cytotoxic effect of asclepiasterol on MDR cells (MCF-7/ADR, HepG-2/ADM) and their corresponding parental cell lines (MCF-7, HepG-2) using an MTT assay. As shown in Figure 2A and 2B, treatment with increasing concentrations of asclepiasterol between 0 and 10.0 μM for 48h did not inhibit the proliferation of these cells. More than 90% of cells were viable when treated with 5.0 μM of asclepiasterol. In addition, different cell lines, including HEK293, Chang, and LO2 cells, were also included in this study to evaluate the cytotoxicity of asclepiasterol on non-tumor cells. Asclepiasterol showed no obvious cytotoxic effect on these cells (Figure 2C). These results suggested that asclepiasterol was not cytotoxic, making it a candidate compound to investigate its MDR activity. Based on the above results, asclepiasterol at concentrations of 2.5 μM and 5.0 μM were used in subsequent experiments.

### Asclepiasterol reverses P-gp-mediated MDR *in vitro*

We next investigated asclepiasterol's modulating effect on MDR cells and the corresponding parental cell lines using an MTT assay. The IC_50_values of chemotherapeutic agents, including Dox, EPI, DNR, paclitaxel and cisplatin, for sensitive and resistant cells in the presence or absence of asclepiasterol are shown in Table 1. Verapamil, a specific P-gp inhibitor at 10.0 μM was used in this study as a positive control. Asclepiasterol at 2.5 and 5.0 μM enhanced the cytotoxicity of these chemotherapeutic agents in a dose-dependent manner in MDR cells. Asclepiasterol at 5.0 μM showed 35.06-, 16.67-, 167.31-, 285.71-fold, and 62.41-, 33.48-, 24.29-, 85.09-fold, for the reversal of resistance to Dox, EPI, DNR and paclitaxel in MCF-7/ADR and HepG-2/ADM cells, respectively. These effects were stronger than those of 10.0 μM verapamil (the corresponding values were 34.71-, 5.03-, 58.39-, 11.79-fold and 38.44-, 7.43-, 56.81-, 78.11-fold for MCF-7/ADR and HepG-2/ADM, respectively). These results indicated that asclepiasterol specifically reversed P-gp-mediated MDR, and in most cases, asclepiasterol demonstrated stronger reversal effects than the positive control.

### Asclepiasterol enhances the anticancer efficacy of chemotherapeutic agents in MDR cells

**Figure 3:**
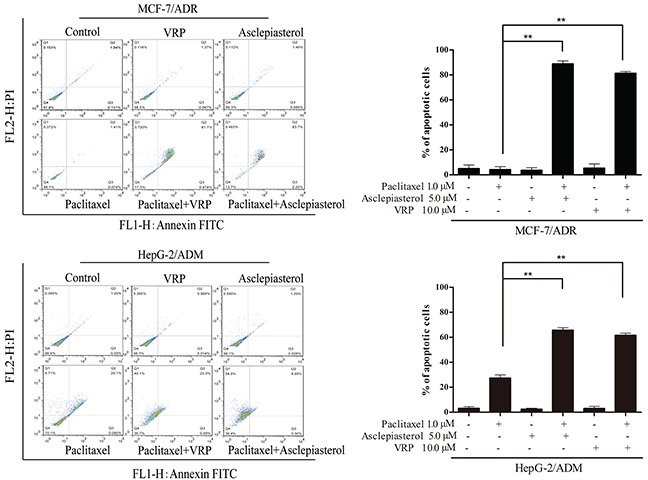
Asclepiasterol sensitizes MDR cells to apoptosis MDR cells were treated with paclitaxel alone (1.0 μM), asclepiasterol alone (5.0 μM), VRP alone (10.0 μM), or their combination for 48h. The IC_50_ values for paclitaxel had been previously determined for each cell line. Apoptosis was analyzed by flow cytometry as the percentage of cells labeled by Annexin V and PI. A representative set of data from three independent experiments is shown. MDR cells were resistant to paclitaxel, and asclepiasterol or VRP increased cell apoptosis induced by paclitaxel. All experiments were repeated three times and data are presented in histogram as means±SD. *P < 0.05, **P < 0.01, compared with doxorubicin treatment alone.

Based on the above results, we speculated that MDR cells might be more sensitive to combination treatment. To examine whether asclepiasterol in combination with the P-gp substrate anticancer agents had any influence on the chemotherapeutic agents mediated apoptosis, we treated MCF-7/ADR and HepG-2/ADM cells with (+) or without (−) 5.0 μM asclepiasterol or 10.0 μM VRP combined with 1.0 μM paclitaxel for 48h, and then measured the extent of apoptosis. Since the IC_50_ values for paclitaxel had been previously determined for each cell line and the MDR cells were resistant to paclitaxel at more than 1.0 μM, paclitaxel in this experiment was tested at 1.0 μM. As shown in Figure 3, treatment of the MDR cells with 1.0 μM paclitaxel, asclepiasterol, or 10.0 μM VRP alone did not cause any apoptosis, while the apoptosis rate of the combination group was dramatically increased as compared with the control group or the treatment alone group (88.93%±2.34% and 81.33%±4.15% for asclepiasterol and VRP combination with paclitaxel, respectively, versus 4.2%±2.35% for paclitaxel alone in MCF-7/ADR and 65.77%±1.92% and 61.6%±1.7% for asclepiasterol and VRP combination with paclitaxel, respectively, versus 27.4%±2.5% for paclitaxel alone in HepG-2/ADM). In contrast, asclepiasterol could not increase paclitaxel-induced apoptosis in sensitive cells (Supplementary Figure S3, supporting information).

**Figure 4:**
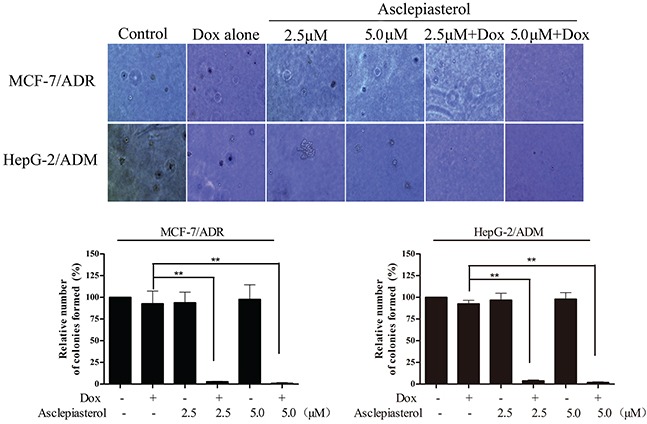
Colony formation assay of MDR cells treated with Dox (3.0 μM) in the absence or presence of Asclepiasterol (2.5, 5.0 μM) Cells were re-suspended and plated in 6-well plates containing DMEM plus 10% FBS in 0.4% agar above a layer of 0.6% agar at a density of 10000 cells per well. Colonies were counted under a phase contrast microscope after 14 days. Representative images of colonies (magnification ×20) are shown. Summary of colony formation assay data from three independent experiments is shown. *P < 0.05, **P < 0.01, compared with doxorubicin treatment alone.

Since the MDR cells with higher P-gp expression were reported to be more malignant and resistant to traditional chemotherapeutic agents [35, 36], we investigated the colony formation capacity of MCF-7/ADR and HepG-2/ADM treated with 3.0 μM Dox and its combination with asclepiasterol by soft agar assay. In this experiment, the dose of Dox was selected according to the cell growth assay and the MDR cells were shown to be resistant at this concentration (Table 1). As expected, the number of colonies formed in MCF-7/ADR and HepG-2/ADM cells was larger than those in the corresponding parental cells, suggesting that the MDR cells were more resistant to Dox treatment (Figure 4). Moreover, no difference was observed in colony size or number between the two MDR cells treated with asclepiasterol alone. In contrast, the combination of asclepiasterol with Dox decreased anchorage-independent cell growth capacity (Figure 4). The number of colonies formed in MCF-7/ADR and HepG-2/ADM were decreased 99.05% and 98.11%, respectively in the combination group. Taken together, these results indicated that asclepiasterol enhanced the efficacy of anticancer agents on the colony formation of MDR cancer cells. However, asclepiasterol did not influence the efficacy of Dox on colony formation in sensitive cells (Supplementary Figure S4, supporting information).

### Asclepiasterol increases the accumulation of Dox and Rh123 in MDR cells

**Figure 5:**
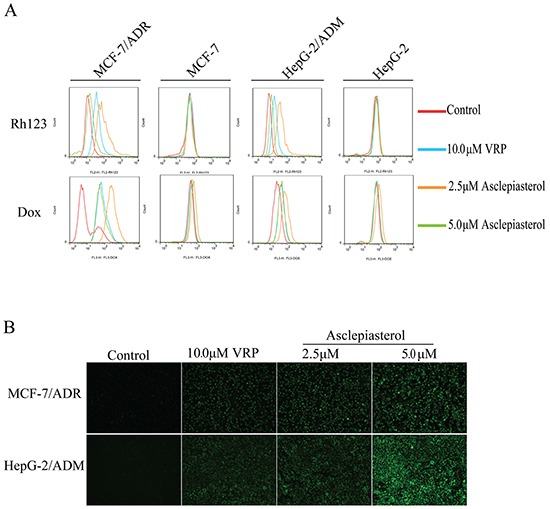
Effect of asclepiasterol at different concentrations on the accumulation of doxorubicin and Rh123 VRP at 10.0 μM was used as positive control. **A.** Flow cytometry analysis of the accumulation of Dox (10.0 μM) and Rh123 (10.0 μM) in MDR cells and their parental cells with or without asclepiasterol or VRP treatment. Intracellular fluorescence was analyzed by flow cytometry. **B.** Fluorescence microscope detection of the accumulation of rhodamine123 (Rh123) in MDR cells after asclepiasterol treatment. Cells were treated with 2.5, 5.0 μM asclepiasterol and 10.0 μM VRP for 48h, and then Rh123 at 10.0 μM was added and incubated for an additional 2h in the dark. Images were taken at 488 nm extraction and 535 nm emission wavelength after washing the cells with cold PBS three times.

The above results showed that asclepiasterol re-sensitized the overexpressing P-gp cells to chemotherapeutic agents which were P-gp substrates. We further examined the effect of asclepiasterol on Dox and Rh123 accumulation in P-gp overexpressing MCF-7/ADR and HepG-2/ADM cells and their corresponding parental cells, MCF-7 and HepG-2. After treatment with (+) or without (−) 2.5 and 5.0 μM asclepiasterol or 10.0 μM VRP (positive control), the cells were analyzed by flow cytometry. As shown in Figure 5A, both MDR cells treated with asclepiasterol accumulated more Dox in a dose-dependent manner as compared with treatment with Dox alone. Intracellular accumulation of Dox was increased by 9.58-, 37.54-, 8.40-fold in MCF-7/ADR, and 1.68-, 3.29-, 1.72-fold in HepG-2/ADM cells in the presence of 2.5 μM and 5.0 μM of asclepiasterol and 10.0 μM VRP, respectively (Figure 5A).

We also tested the effect of asclepiasterol on accumulation of Rh123, a specific P-gp substrate. The fluorescence value of Rh123 was enhanced by 2.98-, 7.46- and 1.20-fold in MCF-7/ADR, and 2.23-, 4.75- and 1.39-fold in HepG-2/ADM cells in the presence of 2.5 μM and 5.0 μM of asclepiasterol and 10.0 μM VRP, respectively (Figure 5B). In contrast, the intracellular accumulation of Dox and Rh123 were not altered in the sensitive cells in the presence of asclepiasterol alone (Supplementary Figure S5, supporting information). We also measured the accumulation of Rh123 in MDR cells treated with asclepiasterol or VRP using fluorescence microscopy and found, consistent with the flow cytometry results, that the MDR cells treated with asclepiasterol or VRP had much higher cytosolic accumulation of Rh123 than the control group (Figure 5B). Both experimental results suggested that asclepiasterol promoted the accumulation of P-gp substrates in MDR cells and therefore re-sensitized them to chemotherapeutic agents.

### Asclepiasterol decreases the expression of P-gp without stimulating or suppressing *MDR1* mRNA expression

**Figure 6:**
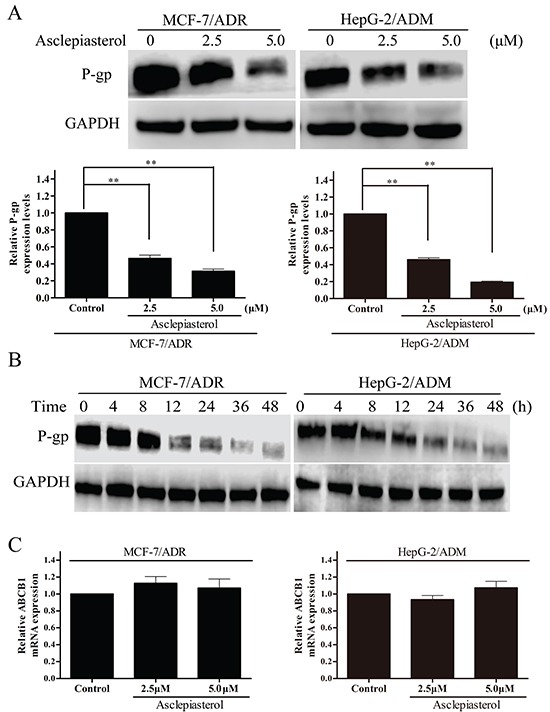
The effect of asclepiasterol on P-gp protein and *MDR1* mRNA expression in MDR cells **A.** MDR cells were treated with asclepiasterol at concentrations of 2.5 and 5.0 μM for 48h. P-gp expression was analyzed by Western blotting. GAPDH was used as a loading control. The summary from three independent experiments is shown under the representative blot image. **B.** Western blot analysis of P-gp expression after a time-course of asclepiasterol treatment. MDR cells were treated with 5.0 μM asclepiasterol for 4 to 48h, P-gp expression was analyzed by Western blotting. GAPDH was used as a loading control. **C.** RT-PCR analysis of *MDR1* mRNA expression in MDR cells after treatment with asclepiasterol at 2.5 and 5.0 μM for 48h. Total RNA was extracted from the cells and the mRNA levels of *MDR1* and *GAPDH* were analyzed by RT-PCR. Independent experiments were performed three times and the summary of the results shown. *P < 0.05, **P < 0.01, compared with the untreated control.

Reversal of P-gp-mediated MDR could be achieved by down-regulating P-gp expression in MDR cells apart from inhibiting its transport function. We therefore measured P-gp expression in MCF-7/ADR and HepG-2/ADM cells before and after asclepiasterol (2.5, 5.0 μM) treatment by Western blot analysis. As shown in Figure 6A, the expression of P-gp in MCF-7/ADR and HepG-2/ADM cells decreased in a dose-dependent manner by 4-7 fold in the presence of 2.5 or 5.0 μM asclepiasterol. We also examined the timing of asclepiasterol-mediated P-gp down-regulation. MCF-7/ADR and HepG-2/ADM cells were treated with 5.0 μM asclepiasterol for 4 to 48h. As shown in Figure 6B, P-gp expression began to decrease at 12h and this timing did not vary among the cell lines we tested.

We next used RT-PCR to examine *MDR1* mRNA expression in MDR cells in the presence or absence of asclepiasterol under the same conditions used for the Western blot experiment. Total RNA was extracted and RT-PCR was performed using *MDR1* or *GAPDH*-specific oligonucleotides. As shown in Figure 6C, *MDR1* mRNA levels were unchanged after asclepiasterol treatment at all concentrations in each of the MDR cells. From these data, we concluded that the effect of asclepiasterol in reducing the efflux of chemotherapeutic agents might be via the suppression of P-gp translation, since *MDR1* mRNA levels had not been altered by asclepiasterol treatment while the protein level was decreased.

### Asclepiasterol suppresses the phosphorylation of ERK1/2, but does not inhibit the phosphorylation of AKT in MDR cells

**Figure 7:**
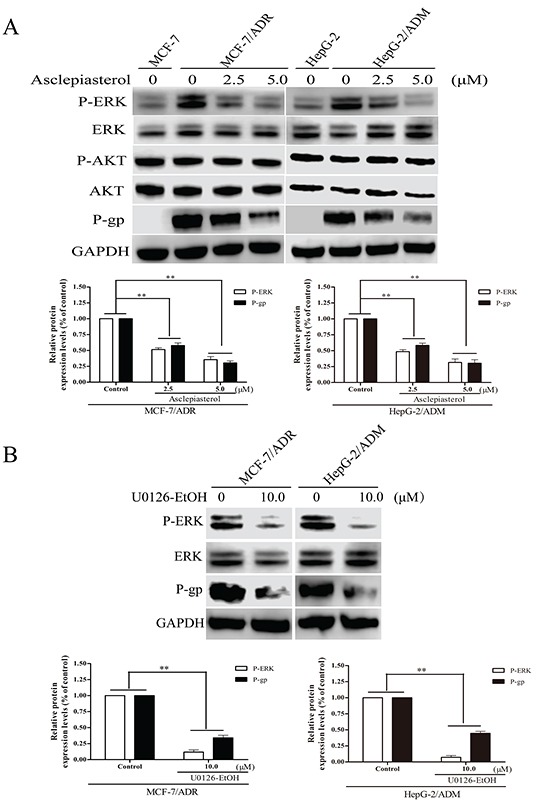
Effect of asclepiasterol on the blockade of ERK1/2 and AKT phosphorylation in MDR cells **A.** Western blot analysis of P-gp expression and ERK1/2, Akt phosphorylation status in MDR cells treated with 2.5 or 5.0 μM asclepiasterol for 48h. GAPDH was used as a loading control. MCF-7 and HepG-2 cells were used as negative controls for detection. **B.** Western blot analysis of P-gp expression and ERK1/2 phosphorylation status in MDR cells treated with 10.0 μM U0126-EtOH for 12h. Relative P-gp and P-ERK expression of MDR cells from three independent experiments are shown under each Western blot image, *P < 0.05, **P < 0.01, compared with the untreated control.

Many groups have reported that aberrant activation of AKT and ERK1/2 signaling pathways is important for the development of the MDR phenotype of tumor cells, and the suppression of ERK/AKT signaling is considered an effective therapeutic approach to overcome MDR [37, 38]. Thus we hypothesized that ERK/AKT pathways might be involved in asclepiasterol-mediated P-gp down-regulation. To test this hypothesis, we determined the total and phosphorylation levels of ERK1/2 and AKT in asclepiasterol-treated cells by Western blot. As shown in Figure 7A, phosphorylated ERK1/2 was increased in the P-gp overexpressing cells MCF-7/ADR and HepG-2/ADM as compared with the corresponding parental cells (MCF-7 and HepG-2). Asclepiasterol treatment led to a reduction in phosphorylated ERK1/2 particularly at 5.0 μM, without stimulating or suppressing the total ERK1/2 levels in the MDR cells. Meanwhile, P-gp expression was decreased in both MDR cells, and associated with the suppression of phosphorylation ERK1/2. However, the concentration of asclepiasterol we used in this experiment did not reduce phosphorylated AKT or total AKT levels in these cells. Taken together, these results suggested that the mechanism of asclepiasterol-mediated P-gp down-regulation was likely through a blockade of the ERK1/2 pathway.

The above results indicated that asclepiasterol could suppress the phosphorylation of ERK1/2 and decrease P-gp expression in the MDR cells at the same time. This suggested that asclepiasterol might reverse P-gp-mediated MDR through the MEK-ERK signaling pathway. Therefore, the MEK1/2 inhibitor, U0126-EtOH, was used to test its effect on P-gp expression in MDR cells. Cells were treated with 10.0 μM U0126-EtOH for 12h and P-gp expression determined by Western blotting. U0126-EtOH suppressed the phosphorylation of ERK1/2, and the MDR cells treated with U0126-EtOH showed 2.94- and 2.25-fold lower levels of P-gp for MCF-7/ADR and HepG-2/ADM, respectively, as compared with the untreated cells (Figure 7B). These results suggested that the MEK-ERK pathway was crucial for the regulation of P-gp expression.

### EGF activates the MEK-ERK pathway and reverses asclepiasterol-mediated P-gp down-regulation in MDR cells

**Figure 8:**
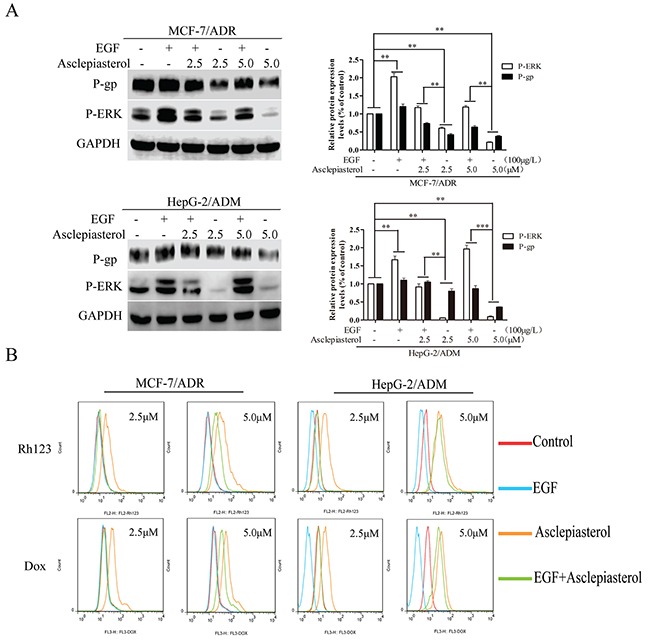
Effect of EGF on asclepiasterol-mediated P-gp down-regulation and P-ERK suppression **A.** Western blot analysis P-gp expression and ERK1/2 phosphorylation after treatment of MDR cells with EGF alone, asclepiasterol alone (2.5, 5.0 μM), or their combination. MDR cells were treated with (+) or without (−) asclepiasterol for 48h to down-regulate P-gp expression on the cell surface and then were treated with (+) or without (−) asclepiasterol combined with 100μg/L EGF for an additional 24h. P-gp and P-ERK1/2 expression were determined by Western blot analysis. GAPDH was used as loading control. Relative P-gp and P-ERK expression of MDR cells from three independent experiments are shown. **B.** Flow cytometry analysis of the accumulation of Dox (10.0 μM) and Rh123 (10.0 μM) in MDR cells treated with asclepiasterol (2.5, 5.0 μM) in combination with 100μg/L EGF. MDR cells were treated under the same conditions as in (A), and were incubated with Dox (10.0 μM) or Rh123 (10.0 μM) for a further 2h. Intracellular fluorescence was analyzed by flow cytometry. *P < 0.05, **P < 0.01, ***P < 0.001 compared with the untreated control.

Given that treatment with asclepiasterol and suppression of P-gp expression in MDR cells was associated with a blockade of the MEK-ERK pathway, we next investigated whether the activation of this pathway would enhance P-gp expression and reverse the effects of asclepiasterol. MCF-7/ADR and HepG-2/ADM cells were treated with (+) or without (−) asclepiasterol for 48h to decrease P-gp expression on the cell surface. Then we treated the cells with (+) or without (−) asclepiasterol combined with 100 μg/L EGF, an activator of the ERK1/2 pathway, for an additional 24h. P-gp and P-ERK1/2 expression were determined by Western blot analysis. The ERK1/2 pathway was activated in the presence of EGF with increased expression of phosphorylated ERK1/2, and the asclepiasterol-mediated down-regulation of phosphorylated ERK1/2 in the two MDR cells was suppressed (Figure 8A). There also was an obvious increase in P-gp expression in these two MDR cells, associated with the increased phosphorylated ERK1/2 expression by EGF.

To further confirm these findings, we next examined whether the effect of asclepiasterol-mediated accumulation of P-gp substrates in MDR cells could be reversed by EGF intervention through treatiment of the MDR cells with asclepiasterol in combination with EGF. As shown in Figure 8B, 100 μg/L EGF could completely block the effect of 2.5 μM asclepiasterol on Dox or Rh123 accumulation in the MDR cells, while those treated with 5.0 μM asclepiasterol were just partially reversed. These results further indicated that the MEK-ERK pathway was involved in asclepiasterol-mediated P-gp down-regulation.

## DISCUSSION

In this study, we identified a novel, natural C_21_ steroidal glycoside, asclepiasterol, from a series of 68 constituents from the milkweed *A. curassavia*, and found it was capable of reversing P-gp-mediated MDR. The natural steroids can be classified into C_18_, C_19_, C_21_, C_23_ (mainly cardenolides with a five-membered lactone ring), C_24_ (mainly bufadienolides with a six-membered lactone ring) and C_27_ structural types which are different in the ring structures and side chain at position C-17 [41]. The P-gp inhibitors identified by de Ravel and Zeino et al. [39, 40] belong to cardiotonic steroid derivatives. In contrast, asclepiasterol is a natural C_21_ steroid, which has been known to possess less toxicity than the cardenolides and bufadienolids [42].

In our preliminary study, we verified the overexpression of P-gp in MCF-7/ADR and HepG-2/ADM cells by Western blotting. Being a candidate reversal modulator, the compound should be within the limits of toxicity acceptable for anticancer treatment [20]. Therefore, asclepiasterol was examined for cytotoxicity in tumor and non-tumor cells. The results indicated that asclepiasterol at concentrations below 10.0 μM did not inhibit cell proliferation; however, it was found to remarkably potentiate the anticancer activity of different chemotherapeutic agents (doxorubin, epirubicin, paxlitaxel and daunorubicin) in the P-gp overexpressing cell lines.

The mechanism of ABC transporter-mediated MDR is that they function in cellular detoxification [43] by pumping a wide range of cytotoxic drugs out of the cancer cells and preventing apoptosis. Many P-gp modulators [44–46] were developed to inhibit this process and make the MDR cells re-sensitize to anticancer agents. With this mechanism in mind, the apoptosis assay was conducted to investigate the potentiation of anticancer activity of paclitaxel by asclepiasterol. A significant increase of apoptosis was observed only for the combination of paclitaxel and asclepiasterol, while asclepiasterol alone showed no influence on cell proliferation. Anchorage-independent soft agar colony growth is a simple but useful assay commonly used to evaluate the malignant and resistant cells responsible for cancer recurrence [47]. As expected, the MDR cells formed more colonies and showed great resistance to Dox treatment; while the number of colonies treated by the combination of asclepiasterol and Dox was significantly reduced. Consistent with these results, asclepiasterol increased the accumulation of Rh123 and Dox in MDR cells in a dose-dependent manner.

P-gp is the most prominent ABC transporter with a diverse spectrum of substrates ranging from 330 Da to 4000 Da [48]. The large amount of data concerning herbal medicines or natural products can be classified into two major categories: functional inhibition of P-gp by interference with efflux activity and down-regulation of P-gp/*MDR1* expression [24]. We found that asclepiasterol could significantly decrease P-gp expression in two MDR cells. Since P-gp has been reported to have a long half-life [18], we therefore examined the time-dependency of asclepiasterol-mediated P-gp down-regulation, and found that the expression of P-gp began to decrease at 12h in MCF-7/ADR and HepG-2/ADM cells, which was consistent with a previous report [38]. To the best of our knowledge, this study was the first report demonstrating the down-regulation of MDR transporter P-gp by a natural steroidal glycoside. Although asclepiasterol suppressed P-gp expression in two MDR cells, we did not observe significant *MDR1* mRNA alterations under the same conditions.

The regulation of ABC transporter expression, e.g. P-gp and BCRP, are very complicated [38, 49]. Some groups have reported that the stability of P-gp is correlated with the ubiquitin-proteasome system [50, 51], which is crucial for regulating intracellular protein turnover, and thus modulating the ubiquitination of P-gp might be a novel approach for the reversal of MDR [49, 52]. In recent years, the relationship between ABC transporter expression and cell growth signaling pathways, especially PI3K/AKT and MAPK/ERK, has received attention. For example, the expression of *BCRP* was correlated with AKT signaling [53], while Katayama et al. [38] reported that the inhibition of MAPK/ERK pathway resulted in the suppression of P-gp. Our results showed that the MAPK/ERK signaling pathway was activated in the overexpressing P-gp MDR cells (MCF-7/ADR, HepG-2/ADM) as compared to their parental cells (MCF-7, HepG-2). The activation states, however, could be inhibited by asclepiasterol treatment via reduction of P-ERK1/2 expression, and the expression of P-gp was also decreased by asclepiasterol in a dose-dependent manner. These results suggested that asclepiasterol might reverse P-gp-mediated MDR through the P-ERK signaling pathway. Therefore, U0126-EtOH, a MEK inhibitor, was used and found to decrease P-gp expression.

In contrast, asclepiasterol treatment for 48h did not change total AKT protein or phosphorylated AKT levels in MDR cells. These results suggested that the expression of P-gp might be regulated by the MAPK/ERK pathway, and that the kinase activities of this pathway are necessary for P-gp stabilization. In order to further confirm the involvement of the MAPK/ERK pathway in the regulation of P-gp, we examined P-gp expression in MDR cells treated with EGF, an activator of the MAPK/ERK pathway. As expected, stimulation of EGF activated the MAPK/ERK pathway by enhancing the P-ERK1/2 expression, and asclepiasterol-mediated P-gp down-regulation was inhibited by EGF. Furthermore, we found that intracellular Dox and Rh123 levels were increased after asclepiasterol treatment for 48h, and this effect could be inhibited by EGF after treatment for another 24h. These results further supported that P-gp expression in MDR cells was positively regulated by the MAPK/ERK signaling pathway.

It is noteworthy that P-gp expression began to change at 12h in MDR cells after asclepiasterol treatment; however, increased accumulation of Rh123 could be observed at 4h prior to the down-regulation of P-gp. A probable explanation for this phenomenon is that asclepiasterol could also interfere with P-gp's function. To test this hypothesis, we have examined the effects of asclepiasterol on the function of *P-gp* using the P-gp ATPase assay [54], and found that asclepiasterol at 5.0 μM inhibits P-gp ATPase activity in a similar manner as Na_3_VO_4_. The details regarding the interactions of asclepiasterol with P-gp and the subsequent pathway will be reported elsewhere.

However, there are still some issues remaining unclear in our study. First, how did asclepiasterol decrease the expression of P-gp while mRNA expression was not changed? We suspect that ubiquitination and proteasomal degradation pathways might be involved. Second, since we showed that inhibition of the MAPK/ERK pathway by asclepiasterol suppressed P-gp expression, we need to further elucidate what factors are involved in this mechanism. Lipid rafts have been reported to be associated with different growth factor and receptor signaling [55–57], which are necessary for P-gp function and expression [58]. Thus, molecular interactions between asclepiasterol and lipid rafts might be a solution to figure out the intrinsic factors involved in the MAPK/ERK mediated P-gp expression. Third, can asclepiasterol reverse P-gp-mediated MDR *in vivo*? Due to the limited amount of asclepiasterol available, only *in vitro* studies were performed. Chemical synthesis will provide large quantities necessary for *in vivo* studies. Fourth, as a MDR reversal candidate agent, we focus on the pharmacodynamic effects and the underlying mechanism of asclepiasterol while the effect of this compound on the pharmacokinetics of conventional chemotherapeutic agent remains unclear.

In conclusion, we found that asclepiasterol, a natural C_21_ steroid glycoside from *A. curassavica* can reverse P-gp-mediated MDR by suppressing the expression of P-gp and therefore increasing the intracellular accumulation of anticancer agents in P-gp-overexpressing cells. Furthermore, we also showed that the asclepiasterol-mediated P-gp down-regulation was associated with a blockade of the MAPK/ERK pathway. Our data suggest that asclepiasterol could be a potential candidate for the reversal of P-gp-mediated MDR. So far, at least three generations of *MDR1* inhibitors have been reported. Asclepiasterol bears a novel chemical structure whose skeleton is different from the previously reported MDR reversing agents. We might classify asclepiasterol into the fourth generation of *MDR1* inhibitors according to recent reports [59].

## MATERIALS AND METHODS

### Isolation and identification of asclepiasterol from *A. curassavia*

Asclepiasterol (scheme 1) was isolated from the ethyl acetate soluble fraction of 70% ethanol extract of the whole plant *A. curassavica* through silica gel column chromatography followed by preparative HPLC. The structure was ambiguously identified as a novel C_21_ steroidal glycoside by a comprehensive analysis of the spectral data (UV, IR, MS, 1D and 2D-NMR). The detailed isolation and structural elucidation are shown in the supporting information (Supplementary Table S1 and Supplementary Figure S1).

### Chemicals and reagents

Asclepiasterol was dissolved in dimethylsulfoxide (DMSO, Sigma Aldrich St. Louis MO) to make a 10 mmol/L stock solution and stored at −20°C. The purity of this compound was analyzed by high performance liquid chromatography and was found to be higher than 99%. The chemical structure was characterized by LC-MS and NMR.

Verapamil (VRP), paclitaxel, MTT, and rhodamine 123 (Rh123) were purchased from Sigma-Aldrich (Deisenhofer, Germany). Doxorubicin (Dox) and epirubicin (EPI) were obtained from Zhe-Jiang HISUN Pharmaceuticals Co (Zhejiang, China). Daunorubicin was supplied by National Institute for the Food and Drug Control (Beijing, China). Antibodies against P-gp/ABCB1, total AKT, total ERK, P-AKT^(Thr 308/Ser473)^ and P-ERK^(Tyr204)^ were products of Cell Signaling Technology. Alexa Fluor 488 goat anti-rabbit IgG (H+L) was purchased from Life Technologies. U0126-EtOH(2,3-bis(amino(2-aminophenylthio)methylene)succinonitrile,ethanol) was obtained from Selleckchem (Houston, TX) and dissolved in DMSO to make a 10μmol/L stock solution and stored at −20°.

### Cell lines and cell culture

The breast cancer cell line MCF-7, Human hepatocellular carcinoma cell line HepG-2, and their Dox-selected P-glycoprotein (P-gp) overexpressing cells MCF-7/ADR and HepG-2/ADM were kindly provided by Cancer institute & Hospital Chinese Academy of Medical sciences (Beijing, China). HEK293, Chang, LO_2_ cells were obtained from the Cell Bank of the Chinese Academy of Sciences (Shanghai, China). All cell lines were cultured in Gibco^®^ RPIM media 1640 or DMEM supplemented with 10% fetal bovine serum, penicillin (100U/mL) and streptomycin (100 mg/ml) and were grown as monolayer cultures and maintained in a humidified atmosphere containing 5% CO_2_ in air at 37°C. Penicillin, streptomycin, RPMI media 1640, and FBS were purchased from Life Technologies, Inc. (Grand Island, NY).

### Cell proliferation assay

Cell proliferation was assessed by MTT assay. In brief, MCF-7, HepG-2, MCF-7/ADR, HepG-2/ADM, HEK293, Chang and LO_2_ cells were seeded in 96-well plates at a density of 4×10^3^ cells per well and cultured overnight. Then cells were treated with various concentrations (0.005-50 μM) of doxorubicin, epirubicin, daunorubicin and paclitaxel in the presence or absence of asclepiasterol or VRP for 48h. 20μL MTT was then added into each well and the cells were incubated for an additional 4h. Finally, the purple formazan crystals formed were dissolved in 150 μL of DMSO and the absorbance was detected at 490 nm by multi-mode microplate reader (Bio-Rad laboratories, USA). The concentration required to inhibit cell growth by 50% (IC_50_) was calculated by using Graph Pad Prism 5.0.

### Dox and Rh123 accumulation assay

Cells were seeded in the six-well plate at a density of 1×10^5^ cells per well and allowed to adhere overnight. Then the cells were treated with two concentrations of asclepiasterol (2.50 μM, 5.0 μM) or 10.0 μM VRP as positive control based on the previous assay for 48h at 37°C before incubating with 10.0 μM Dox or 10.0 μM Rh123 for an additional 2h in darkness at 37°C. After that, cells were harvested and washed 3 times with cold PBS. Finally, cells were re-suspended in PBS buffer and analyzed by Flow cytometry (BD Bioscience, San Jose, CA). Data were analyzed with FlowJo 7.6.1 software.

To further visualize the effect of asclepiasterol on the intracellular retention of Rh123, 5×10^5^ cells per well were seeded in the 6-well plate for overnight. The cells were treated with asclepiasterol (2.5, 5.0 μM) or 10.0 μM VRP for 48h, and were then incubated with 10.0 μM Rh123 alone or 10.0 μM Rh123 combination with asclepiasterol or VRP in the fresh RPMI 1640 medium for 2h in darkness at 37°C. After that cells were washed 3 times with cold PBS and images were acquired by fluorescence microscopy.

### Annexin V/PI staining

For detection of apoptotic cells, HepG-2/ADM and MCF-7/ADR cells were treated with (+) or without (−) 2.5 or 5.0 μM asclepiasterol combined with 1.0 μM paclitaxel for 48h. Cells were then harvested and washed 3 times with binging buffer. After that, cells were incubated with fluorescein isothicocyannte (FITC)-conjugated annexin V reagent and PI in binging buffer for 15 min at room temperature as described by the manufacture (BD Bioscience, San Jose, CA) and finally analyzed by Flow cytometry. Data were analyzed with FlowJo 7.6.1 software.

### Soft agar colony formation assay

Soft agar colony formation was assessed according to Cifone and Fidler [60]. In brief, the MDR cells MCF-7/ADR and HepG-2/ADM were treated with asclepiasterol for 24h. Then the cells were re-suspended and plated into six-well plates containing DMEM plus 10% FBS in 0.4% agar above a layer of 0.6% agar at a density of 1000 cells per well. Colonies were counted under a phase contrast microscope after 14 days. All experiments were done in triplicate and repeated three times.

### Western blotting analysis

Western blotting analysis was performed as previously described [61]. Cells (1×10^6^) were incubated with 2.5 or 5.0 μM asclepiasterol for 48h. For immunoblotting, cells were pelleted by centrifugation at 320 ×g for 10 min and suspended in lysis buffer (20 mM Tris-HCl pH 7.4, 2 mM EDTA, 500 mM sodium orthovanadate, 1% Triton X-100, 0.1% SDS, 10 mM NaF, 10 mg/mL leupeptin, and 1 mM PMSF). Aliquots (20 mg) of the lysates were separated on a 4–12% SDS-polyacrylamide gel and transferred into a PVDF membrane (Millipore, USA). Blots were blocked for 2 h in blocking buffer (5% non-fat dry milk in PBST buffer (10 mM phosphate buffer, 2.7 mM KCl, 140 mM NaCl and 0.05% Tween 20, pH 7.4)) and incubated with primary antibodies (1:1000) overnight at 4°C. After washing with PBST, the appropriate HRP-conjugated secondary antibody (1:2000) was added to the preparation. The blot was incubated at 37°C for 1 h and developed using an enhanced chemiluminescence detection kit (Beyotime Institute of Biotechnology, China).

### Real-time PCR

*MDR-1* mRNA expression was examined by RT-PCR. Cells were treated with asclepiasterol under the same condition used for Western blot analysis. Total cellular RNA was extracted by using the Trizol reagent (Takara, Japan). Complementary DNA (cDNA) corresponding to 0.8 μg of total RNA was used for per reaction (20μL) in a real-time quantitative PCR reaction performed on a Roche Light cycler (Manncheim, Germany) using power SYBER Green Master Mix (Takara, Japan).

### Statistical analysis

Each experiment was performed at least three times and the data were presented as mean ± standard deviation (SD). Statistical significance of the results was calculated by one-way analysis of variance (ANOVA) or two-way ANOVA using Prism 5 (GraphPad, La Jolla, CA) and the prior significance level was set at *P* < 0.05, *P* <0.01, *P* <0.001.

The multidrug resistance ratio (MR) was defined to evaluate the extent of cell resistance to the anti-cancer drugs:

MR = *IC*_50 (the P-gp overexpressing cell lines)_/*IC*_50 (the corresponding parental cell lines)_

The reversal ratio (RR) was defined to evaluate the ability of a reversing agent to reverse the MDR:

RR = *IC*_50 (the P-gp overexpressing cell lines)_/*IC*_50 (the presence of reverser the P-gp overexpressing cell lines)_

## SUPPLEMENTARY FIGURES AND TABLE


