# Supplementary material for: Asclepiasterol, a novel C21 steroidal glycoside derived from Asclepias curassavica, reverses tumor multidrug resistance by down-regulating P-glycoprotein expression
Source: Oncotarget. 2016 Apr 25;7(21):31466–83. doi: 10.18632/oncotarget.8965 (PMC5058771; doi:10.18632/oncotarget.8965)
Supplement: Supplementary file 1 [file oncotarget-07-31466-s001.pdf]

Asclepiasterol, a novel C_{21} steroidal glycoside derived from *Asclepias curassavica*, reverses tumor multidrug resistance by down-regulating P-glycoprotein expression

SUPPLEMENTARY INFORMATION

EXTRACTION, ISOLATION AND IDENTIFICATION OF ASCLEPIASTEROL

General experimental procedure

ESIMS and HRESIMS spectra were obtained on a Finnigan LCQ Advantage Max ion trap mass spectrometer and an Agilent 6210 ESI/TOF mass spectrometer, respectively. Melting points were measured on an X-5 melting point apparatus without correction. Optical rotation was recorded in MeOH on Jasco P-1020 polarimeter at room temperature. Ultraviolet (UV) spectra were determined by V-550 UV/vis spectrophotometer, while IR spectra were collected from a Jasco FTIR-480 Plus spectrometer. Nuclear magnetic resonance (NMR) spectra were obtained on a Bruker AV-300 or AV-400 spectrometer. Thin-layer chromatography (TLC) analyses were carried out using pre-coated silica gel GF_{254} plates (Qingdao Marine Chemical Plant, Qingdao, People's Republic of China). Silica gel for column chromatography (200-300 mesh) was produced by Qingdao Marine Chemical Industrials, and Sephadex LH-20 was purchased from Pharmacia Biotech (Pharmacia, Kalamazoo, MI, USA). Preparative HPLC was performed on a Varian Prostar system equipped with a preparative Cosmosil C_{18} column (20×250 mm) column.

Plant material

The whole plant of *Asclepias curassavica*. was collected from Lvzi endowment landscape nursery stock production base in Guangdong province of China in August, 2012 and authenticated by Professor Guang-Xiong Zhou (Jinan University). A specimen (No. 2012081001) was deposited in the Institute of Traditional Chinese Medicine and Natural Products, College of Pharmacy, Jinan University, P. R. China.

Extraction and isolation

The dried and powdered whole plants of *Asclepias curassavica* (20.0 kg) were extracted with 70% (V/V) ethanol at 90°C three times. The solution was combined and concentrated under reduced pressure to afford a crude extract (3.0 kg), which was suspended in distilled water and partitioned with n-hexane, ethyl acetate, n-butanol. The ethyl acetate soluble fraction (200 g) was subjected to silica gel column chromatography eluted with gradient mixtures of $CHCl_3$-MeOH (100:0→1:4) to yield 18 fractions (Fr. 1 to Fr. 18). Fraction 6 eluted by $CHCl_3$-MeOH (10:1) was further purified by preparative HPLC eluted by CH_3CN-H_2O (7:3) to afford compound asclepiasterol (28 mg, Supplementary Scheme S1),

Asclepiasterol, white powder; $[\alpha]_D^{25}$ -49 (0.1, MeOH); UV (MeOH) λmax (log ε) 203(1.39), 229 (1.36), 273(0.34)nm; IR (KBr) νmax 3463, 2933, 1714, 1637, 1384, 1165, 1060cm^{-1}; ^{1}H and ^{13}C NMR data, see Table S1; HRESIMS *m/z* 927.3624 [M-H]$^-$ (calcd for $C_{50}H_{71}O_{16}$, 927.3624).

Structural identification

Asclepiasterol was obtained as a white amorphous powder. The negative HRESIMS gave an [M−H]$^-$ peak at *m/z* 927.3624, corresponding to the molecular formula $C_{50}H_{72}O_{16}$ (calcd. 927.3624). IR spectrum showed the absorption bands for hydroxyl (3463cm^{-1}), carbonyl (1714cm^{-1}), and olefinic (1637cm^{-1}) groups. The ^{13}C NMR spectrum of asclepiasterolshowed 50 carbon signals, consisting of four olefinic carbons, six aromatic carbons, one aldehyde group and one ester group, as well as six methyls, two methoxyls, ten methylenes, fifteen methines, and five quaternary carbons. Close comparison the NMR (^{1}H, ^{13}C NMR, DEPT, and HMQC, Supplementary Figure S1-1—Supplementary Figure S1-9, supporting information) data of the aglycone of asclepiasterol with those of 12-*O*-(*E*)-cinnamoyltayloron revealed two groups NMR signals including the glycone 12-*O*-(*E*)-cinnamoyltayloron and three sugar moieties because of the presence of three anomeric carbon signals at δ_C 97.0, 102.8 and 100.6 and three corresponding anomeric proton signals at δ_H 4.95 (dd, *J* = 9.4, 1.4 Hz), 4.83 (dd, *J* = 9.9, 1.4 Hz), 4.59 (dd, *J* = 10.2, 1.5 Hz) respectively. NOESY correlations H-1'↔ H-5' and H-4' ↔ H_3-6' (Supplementary Figure S1-10) as well as absence of correlation H-3'↔ H-5' suggested that one of sugar units is β-digitoxopyranose [δ_C: 97.0 (C-1'), 38.9 (C-2'), 68.3 (C-3'), 83.8 (C-4'), 70.0 (C-5') and 18.5 (C-6')]. Similarly, the other two sugar components were assigned to be β-cymarose [δ_C: 100.6 (C-1''), 39.8 (C-2''), 81.6 (C-3''), 69.5 (C-4''), 79.3 (C-5''), 18.5 (C-6'') and 57.4 (OMe), δ_C: 102.8

(C-1’’’), 37.4 (C-2’’’), 78.4 (C-3’’’), 76.9 (C-4’’’), 73.2 (C-5’’’), 18.4 (C-6’’’) and 58.6 (OMe)]. The sugar sequence of asclepiasterolwas determined by the HMBC spectrum (Supplementary Figure S1-10) which showed the correlations between δ_H 4.95 (H-1’) and δ_C 83.6 (C-3), δ_H 4.82 (H-1’’) and δ_C 83.8 (C-4’) and δ_H 4.59 (H-1’’’) and δ_C 69.5 (C-4’’). Therefore, asclepiasterol was deduced to be 12-*O*-(*E*)-cinnamoyltayloron 3-*O*-β-digitoxopyranosyl -(4→1)-β-cymaropyranosyl-(4→1)-β-cymaropyranoside.

SUPPLEMENTARY SCHEME, FIGURES AND TABLE

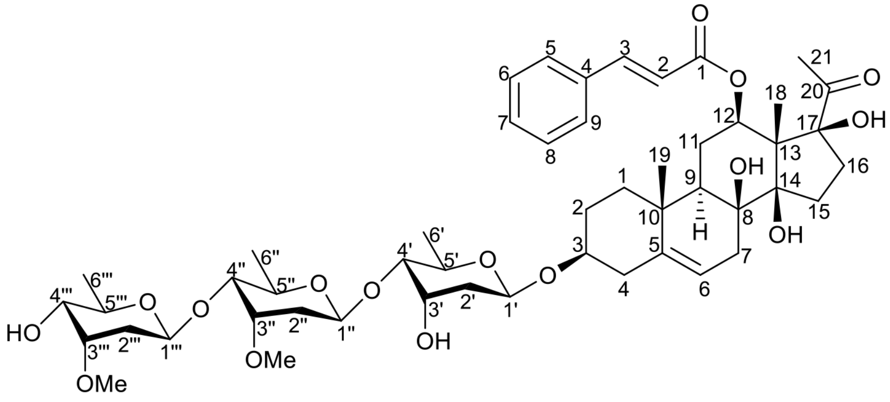

Supplementary Scheme S1: Chemical structure of asclepiasterol.

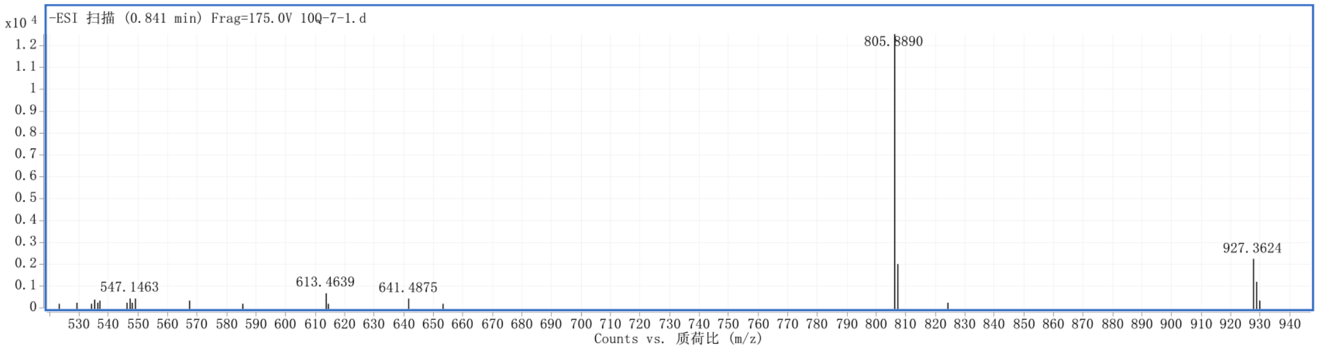

Supplementary Figure S1-1: HR-ESI-MS spectrum of asclepiasterol.

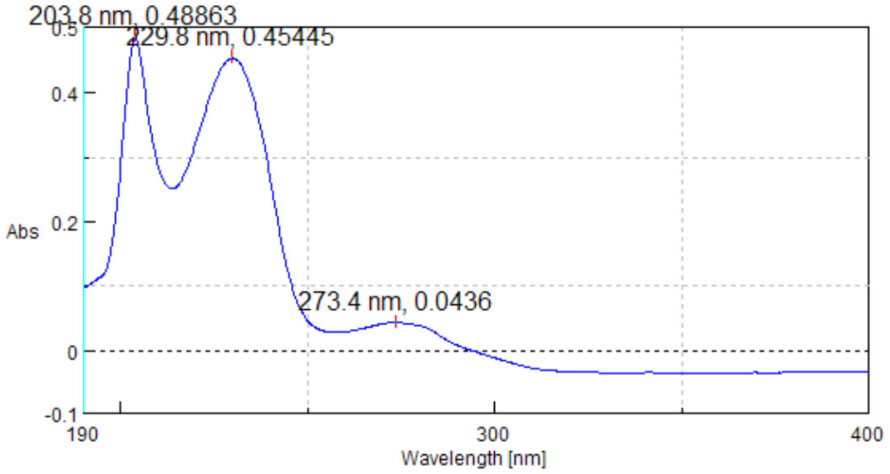

Supplementary Figure S1-2: UV spectrum of asclepiasterol.

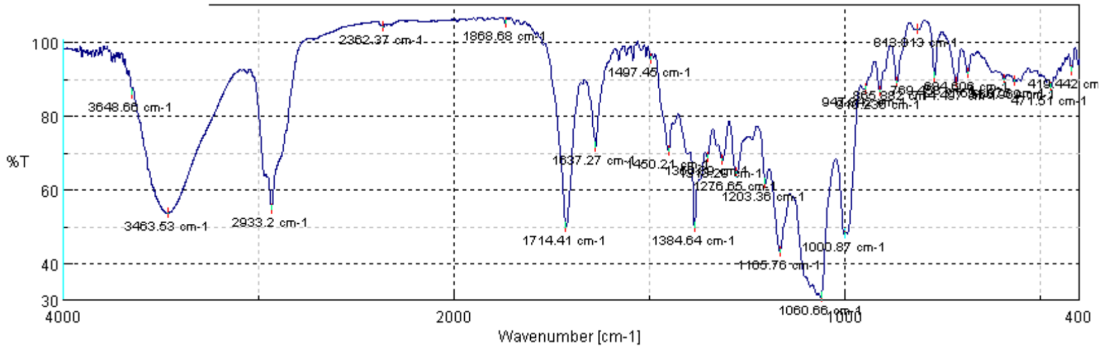

Supplementary Figure S1-3: IR spectrum of asclepiasterol.

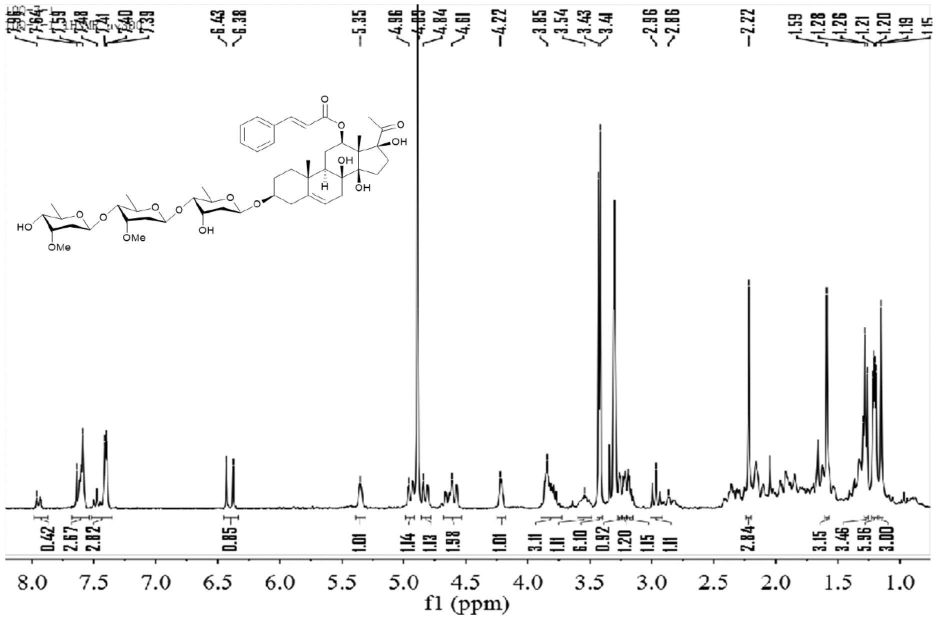

Supplementary Figure S1-4: ^{1}H NMR spectrum of asclepiasterol.

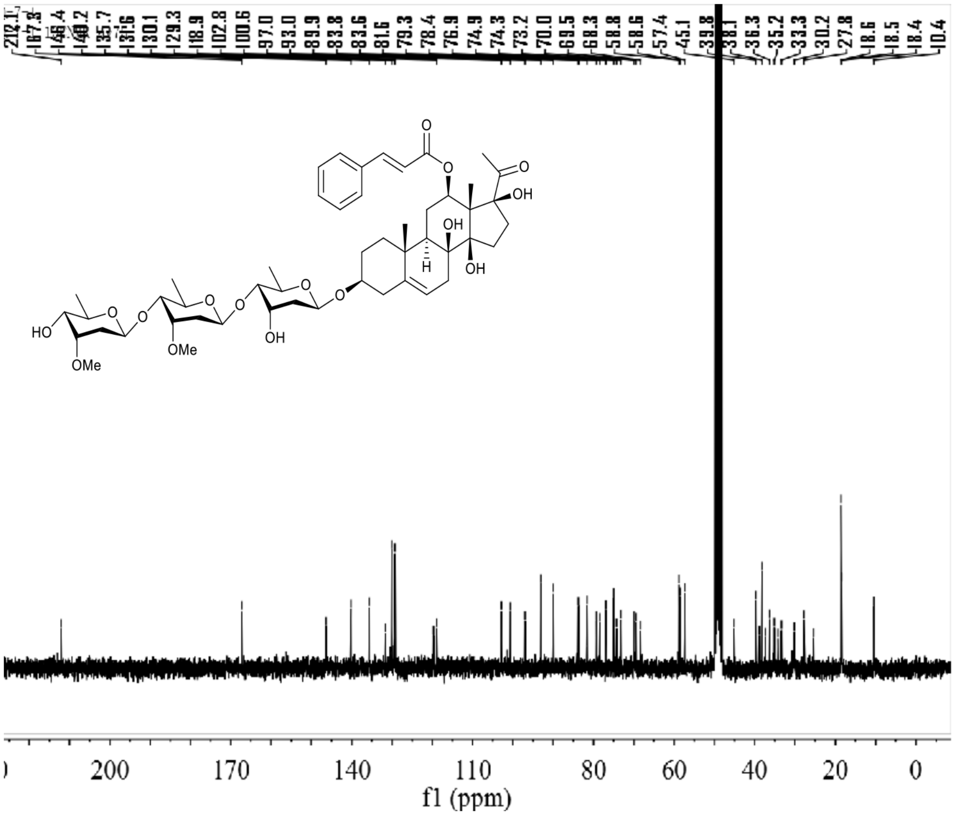

Supplementary Figure S1-5: ^{13}C NMR spectrum of asclepiasterol.

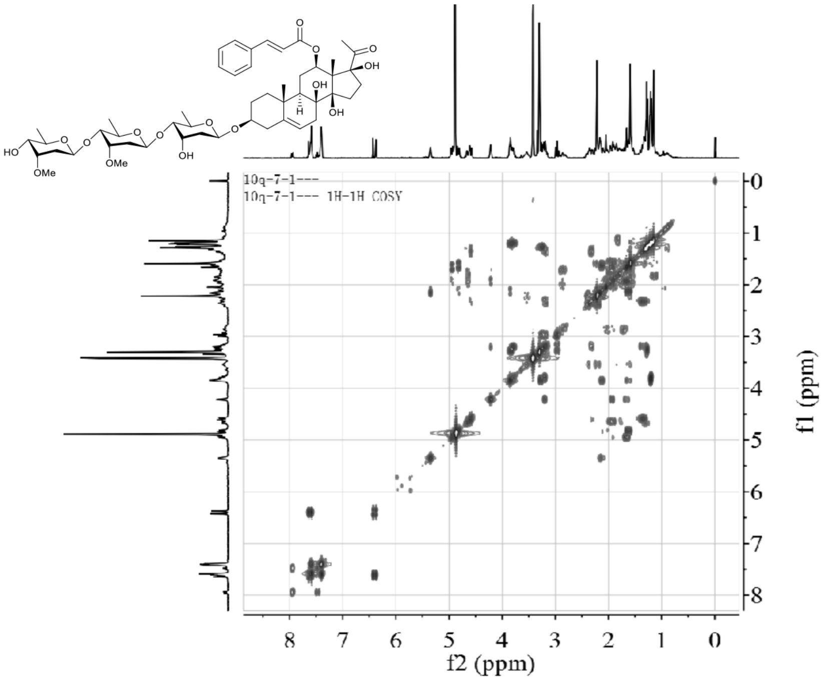

Supplementary Figure S1-6: ^{1}H-^{1}H COSY spectrum of asclepiasterol.

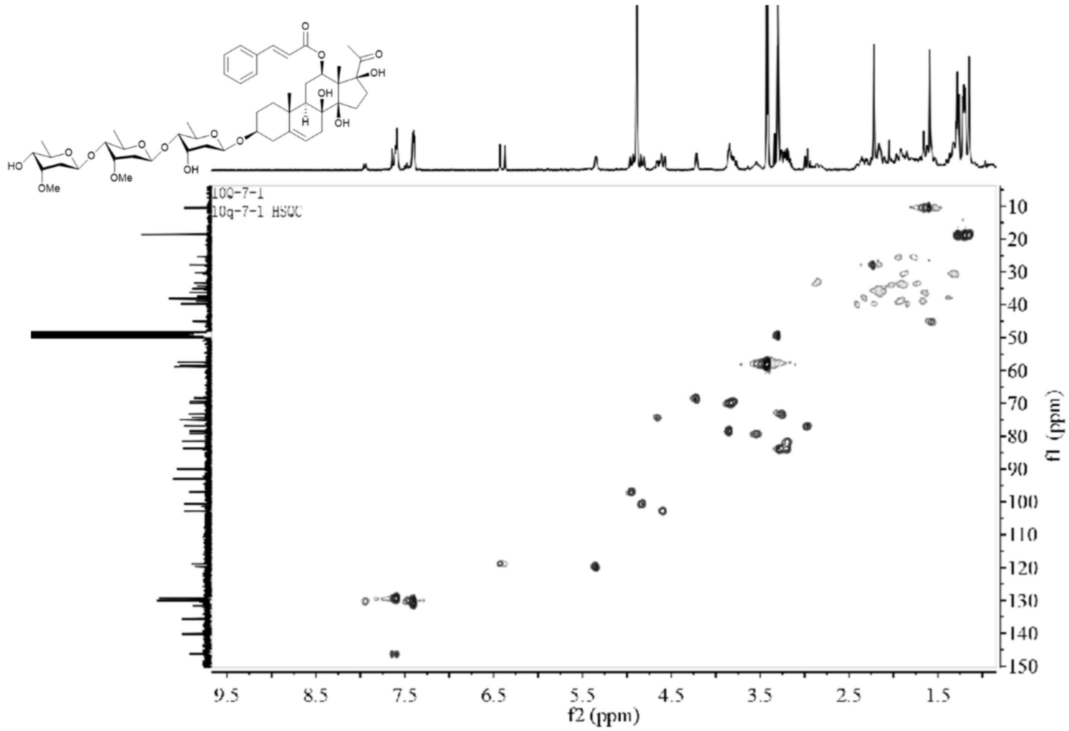

Supplementary Figure S1-7: HSQC spectrum of asclepiasterol.

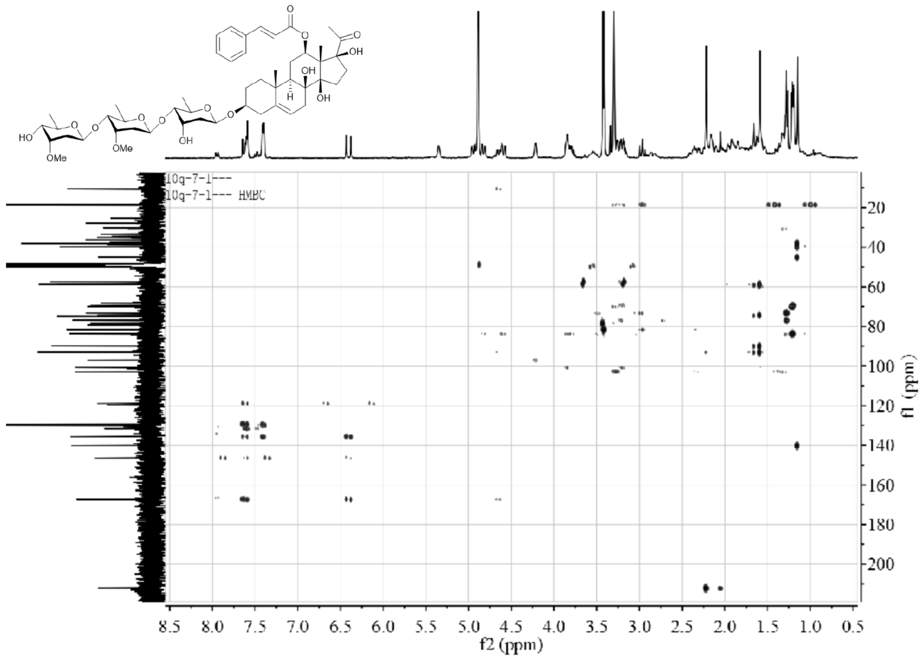

Supplementary Figure S1-8: HMBC spectrum of asclepiasterol.

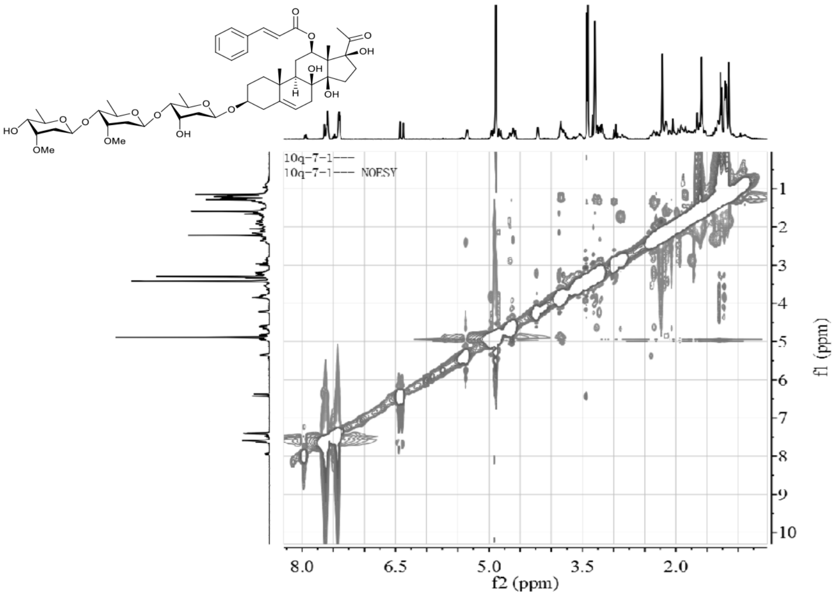

Supplementary Figure S1-9: ROESY spectrum of asclepiasterol.

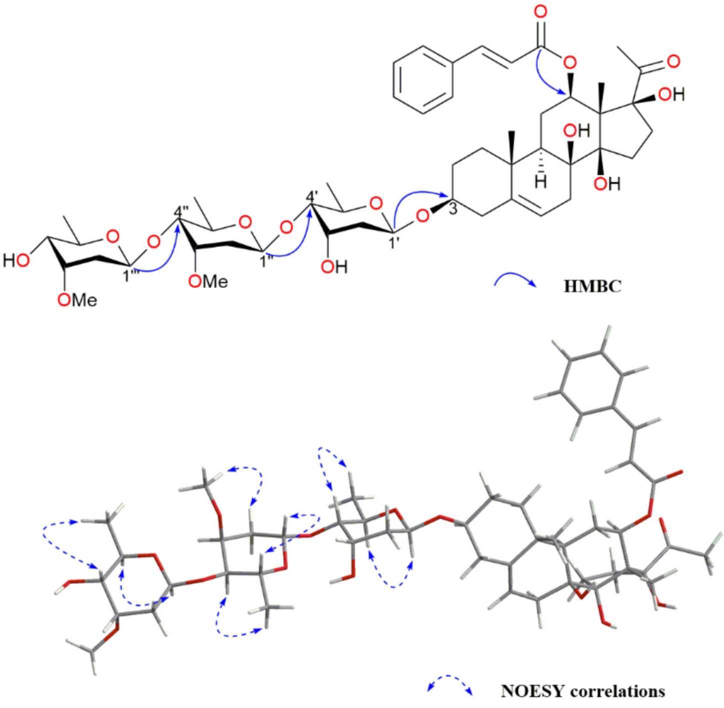

Supplementary Figure S1-10: Key HMBC and NOESY correlations of asclepiasterol.

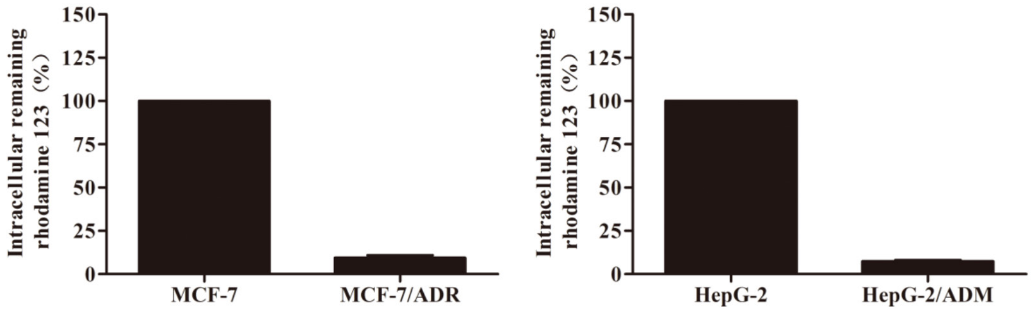

Supplementary Figure S2: The accumulation of Rh123 in MCF-7/ADR and HepG-2/ADM cells in comparison to the corresponding parental cell lines MCF-7 and HepG-2.

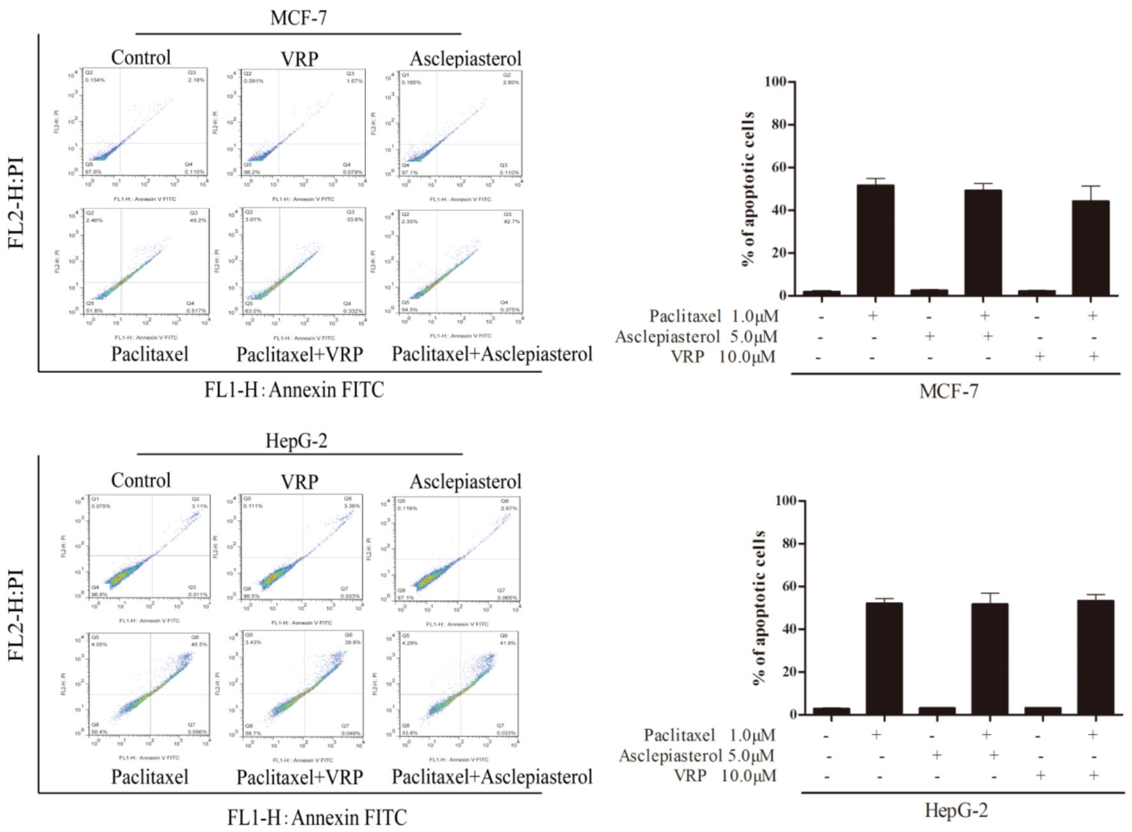

Supplementary Figure S3: The effect of asclepiasterol on the apoptosis of sensitive cells. The sensitive cells were treated with paclitaxel alone (1.0 μM), asclepiasterol alone (5.0 μM), VRP alone (10.0 μM), or their combination for 48h under the same condition as the test of MDR cells. Apoptosis was analyzed by flow cytometry as the percentage of cells labeled by Annexin V and PI. A representative set of data from three independent experiments is shown. All experiments were repeated at thrice and data are presented in histogram as means±SD. **P* < 0.05, ***P* < 0.01, compared with doxorubicin treatment alone.

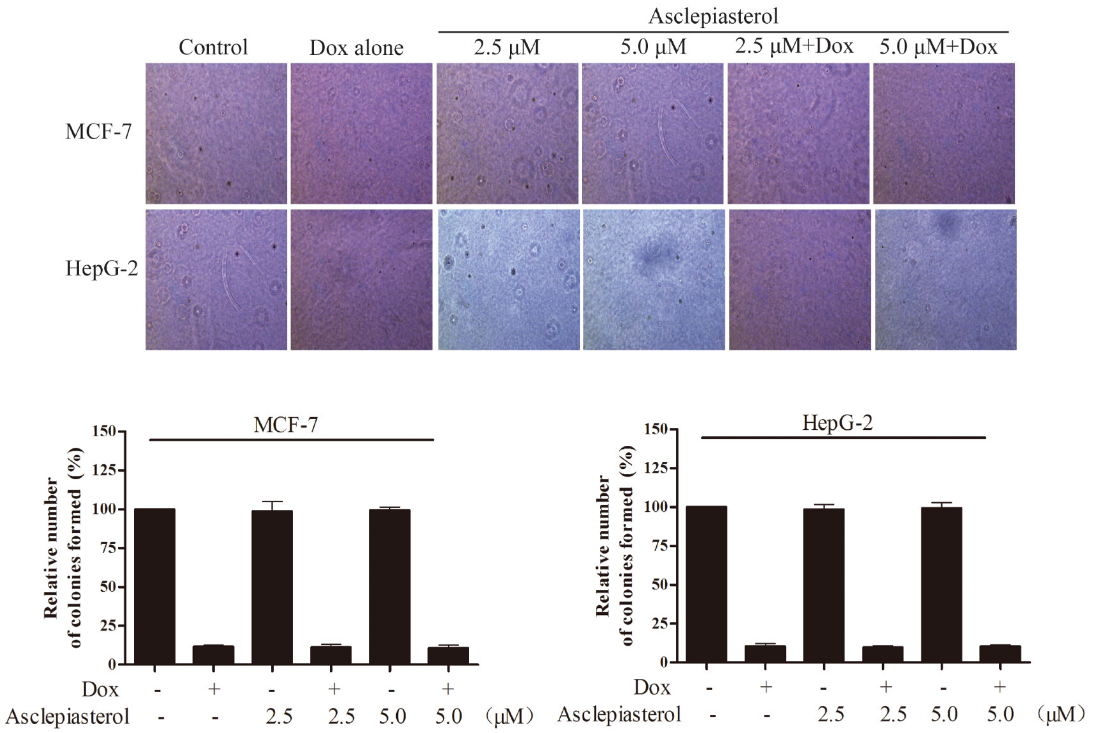

Supplementary Figure S4: Colony formation assay of the sensitive cells (MCF-7 and HepG-2) treated with Dox (3.0 μM) in the absence or presence of asclepiasterol (2.5, 5.0 μM). The cells were re-suspended and plated onto 6-well plates containing DMEM plus 10% FBS in 0.4% agar above a layer of 0.6% agar at a density of 1000 cells per well. Colonies were counted under a phase contrast microscope after 14 days. Representative images of colonies (magnification ×20) and summary of colony formation assay data from three independent experiments were shown. **P* < 0.05, ***P* < 0.01, compared with doxorubicin treatment alone.

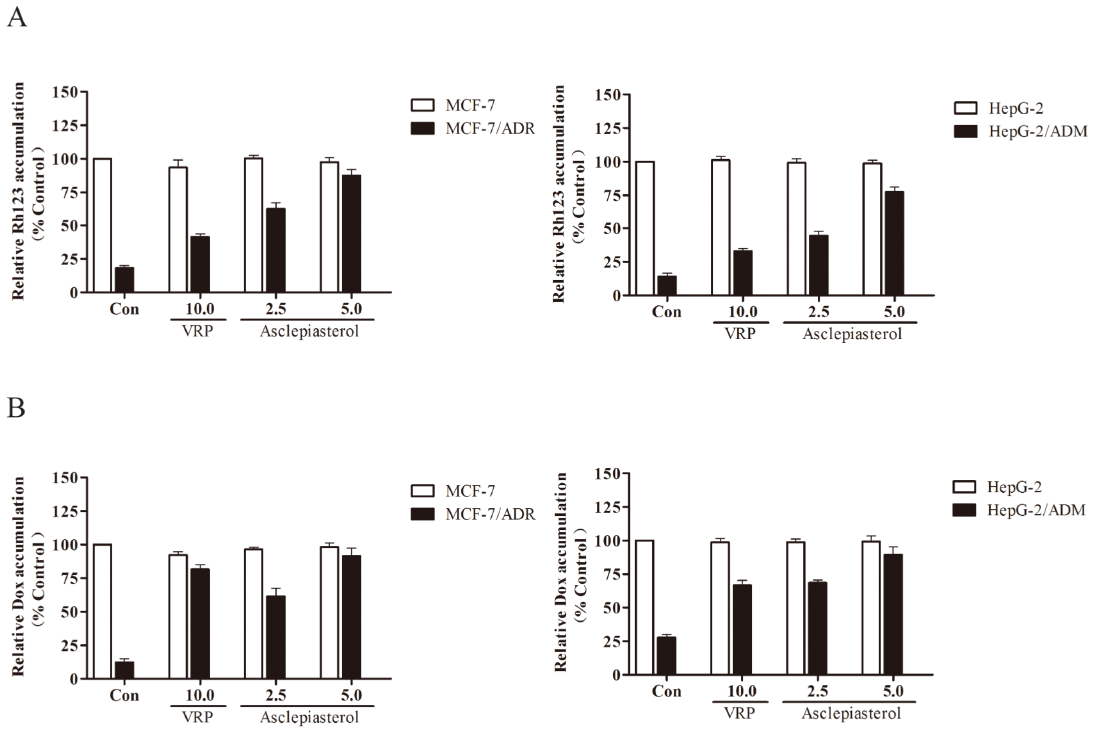

Supplementary Figure S5: The effect of asclepiasterol (2.5, 5μM) on the accumulation of Rh123 and Dox in sensitive and MDR cells. The accumulation of Rh123 and Dox were measured by flow cytometry and the results were analyzed by FlowJo software and calculated by GraphPad. **A.** Summary from three independent experiments of Rh123 accumulation. **B.** Summary from three experiments of Dox accumulation.

Supplementary Table S1: ^{1}H and ^{13}C NMR data for asclepiasterol (δ in ppm J in Hz)

position	δ_C	δ_H (J in Hz)	position	δ_C	δ_H (J in Hz)
1	39.8	1.83 (m), 1.16 (m)	5a	130.1	7.39-7.60 (overlap)
2	30.1	1.87 (m), 1.59 (m)	6a	129.3	7.39-7.60 (overlap)
3	83.6	3.28 (m)	7a	131.6	7.39-7.60 (overlap)
4	36.3	1.63 (m)	8a	129.3	7.39-7.60 (overlap)
5	140.2		9a	130.1	7.39-7.60 (overlap)
6	119.7	5.36 (brs)	Sugar units		
7	35.2	2.18 (m)	1’	97.0	4.95 (dd, 9.6, 1.9)
8	75.0		2’	38.9	1.91 (m), 1.66 (m)
9	45.1	1.59 (m)	3’	68.4	4.22 (dd, 5.7, 2.7)
10	38.2		4’	83.8	3.21 (m)
11	25.5	1.99 (m), 1.81 (m)	5’	70.0	3.82 (m)
12	74.7	4.82 (m)	6’	18.6	1.20 (d, 6.1)
13	59.1		1”	100.6	4.83 (m)
14	90.0		2”	39.8	2.37 (m), 2.21(m)
15	33.5	2.05 (m), 1.74 (m)	3”	81.6	3.19 (m)
16	34.3	2.06 (m), 1.92 (m)	4”	69.5	3.82 (m)
17	93.1		5”	79.3	3.85 (m)
18	10.6	1.66 (s)	6”	18.6	1.21(d, 6.1)
19	18.5	1.15 (s)	OMe	57.4	3.41 (s)
20	212.3		1’’’	102.8	4.59 (m)
21	27.9	2.04 (s)	2’’’	37.4	2.33 (m), 1.36(m)
12-O-Acyl			3’’’	78.4	3.85 (m)
1a	167.3		4’’’	76.9	2.97 (m)
2a	118.9	6.40 (d, 16.1)	5’’’	73.3	3.26 (m)
3a	146.4	7.61 (d, 16.1)	6’’’	18.5	1.27 (d, 6.1)
4a	135.7		OMe	58.5	3.43 (s)

^{1}H and ^{13}C NMR data were recorded on 300 MHz spectrometers in methanol-d_4.
